# Microplastics as Contaminants in Water Bodies and Their Threat to the Aquatic Animals: A Mini-Review

**DOI:** 10.3390/ani12202864

**Published:** 2022-10-20

**Authors:** Mingshi Chen, Yuhua Yue, Xiaoxue Bao, Hui Yu, Yuansheng Tan, Binbin Tong, Suksan Kumkhong, Yingying Yu

**Affiliations:** 1 Guangdong Provincial Key Laboratory of Animal Molecular Design and Precise Breeding, School of Life Science and Engineering, Foshan University, Foshan 528225, China; 2 Sinopharm Group Dezhong (Foshan) Pharmaceutical Co., Ltd., Foshan 528225, China; 3 Department of Animal Science, Faculty of Science and Technology, Muban Chombueng Rajabhat University, 46 Moo 3, Chombueng, Ratchaburi 70150, Thailand

**Keywords:** microplastics, water environment, aquatic animals, toxicity, stress

## Abstract

**Simple Summary:**

Microplastics (MPs, diameter of >5 mm), as an environmental stressor, have become the focus of the problematization of plastics in the environment due to their serious global pollution. The literature on the presence of MPs in the worldwide water environment and stress on the aquatic animals associated with MP pollution were reviewed.

**Abstract:**

Microplastics (MPs), which are particles with a diameter of less than 5 mm, have been extensively studied due to their serious global pollution. Typically, MPs in water originate from terrestrial input. A number of studies have reported the presence of MPs as a stressor in water environments worldwide, and their potential threat to the aquatic animals, affecting the growth, oxidative stress responses, body composition, histopathology, intestinal flora, and immune and reproduction systems. During the plastic degradation process, a large variety of toxic substances are released. MPs have been proposed to be the carriers of toxic chemicals and harmful microorganisms. A study of the literature on MP pollution and stress on the aquatic animals associated with MPs was carried out.

## 1. Introduction

Plastics are produced and used in considerable quantities due to their low cost, excellent mechanical properties, stable chemical properties, and other optimal properties. In 2020, plastics production had reached 367 million tons worldwide [1]. Microplastics (MPs), particles with a diameter of <5 mm, are a significant cause of pollution [2]. These MPs fall into two classes, namely primary and secondary. Primary MPs are small particles from daily necessities, while secondary MPs are generated by the decomposition of large plastics into smaller plastic debris through long-term physical, chemical, and biological processes, which may also generate nanoplastics (NPs, diameter: 1–100 nm) [3,4]. In recent years, MPs have emerged as a significant environmental pollutant and an environmental stressor, owing to their challenging degradation processes [5]. For this reason, the Fifth United Nations Environment Assembly voted for a resolution “to end plastic pollution” [6].

The pollution caused by marine MPs was already reported in the early 1970s [7]. A number of studies have shown that MPs can be ingested and accumulated in aquatic animals to cause stress responses, such as affecting the growth, metabolism, behavior, and reproduction of an animal, and causing oxidative stress in these aquatic species. Furthermore, MPs pose a health hazard to other animals through the food chain. A literature study on the pollution caused by MPs and their effects on health hazard in aquatic animals was carried out.

## 2. The Status of Global Water Pollution Caused by MPs

Currently, MPs are an urgent and growing section of global water pollution. MPs in water mainly originate from terrestrial input. Dumped plastic waste, polystyrene flotation gears heavily used in aquaculture, and plastic devices discarded by the offshore drilling platforms are the leading causes of contamination [8]. Synthetic fibers used in industry, as well as articles of daily use, such as cosmetics and toiletries, also contribute to the pollution associated with MPs [9]. The existing water treatment processes are able to remove a majority of MPs in sewage. However, MPs of smaller particle sizes cannot be filtered through the sewage treatment system. For instance, it has been reported that the concentrations of MPs in two typical large tertiary wastewater treatment plants in Shanghai (China) were 226.27 ± 83.00 and 171.89 ± 62.98 items/L with removal rates of 63.25% and 59.4%, respectively [10]. In Harbin (China), the total removal rate of MPs in two sewage-treatment plants was 77.48% and 84.48%, respectively, where the daily emission of MPs approximately 1.47 × 10^6^ items [11]. Thus, the wastewater treatment plants in China are currently not able to effectively remove MPs from wastewater.

Table 1 illustrates the related number of studies which reported the ubiquitous distribution of MPs as a stressor in global water bodies. In China, MPs have been detected in Dongting Lake, Hong Lake [12], the Three Gorges Reservoir [13], the Yangtze Estuary System [14], Haizhou Bay [15], the Urban Lake of Wuhan [16], Pearl River Estuary, the Guangzhou urban area [17], a pond and rice-crayfish co-culture water and sediments in Jianli, Hubei [18], etc. Other areas of the world include Elbe water and sediments [19], Lake Winnipeg in Canada [20], the Mediterranean [21], the Southern Ocean [22], the Pacific [23,24], the Persian Gulf [25], Chao Phraya [26], the Ems River [27], the Han River [28], South Georgia [29], the Baltic Sea [30], the Caspian Sea [31], Rize [32], Rawal Lake [33], the Terengganu estuary and offshore [34], etc.

The degree of MP pollution is influenced not only by the migration and transformation of MPs, but also by population density, human activities, and the proportion of development within watersheds (rural, urban, and industrial). The abundance of MPs in four estuarine rivers in Chesapeake Bay demonstrated statistically significant positive correlations with population density and proportion of development within watersheds [38]. The dense population and industrial complex of Puebla City might lead to a high abundance of MPs in the downstream section of Atoyac River basin (Mexico) [39]. Other studies reported that the abundance of MPs on the windward beaches of Hawai’i [40] and on the urban and non-use beaches in the coastal areas of Bandar Abbas [41] further supported a similar trend. However, in Zhejiang (China), the concentration of MPs in suburban areas was observed to be 430–540 items/m^3^, while it was 1150–2154 items/m^3^ in the plain countryside and mountainous areas, indicating that there was no correlation between abundance of MPs and serviced population [37]. Furthermore, MP pollution appears to be linked to production modes around the treatment facilities. For example, a plastic production area [42] and a textile industrial area [43] can easily cause serious MP pollution and increase the environmental stress.

The continuous discharge of large quantities of plastics can cause serious environmental challenges, and the aquatic animals will be increasingly exposed to MP stress and inevitably forced to ingest MPs. To make matters worse, MPs will be accumulated through the food chain, causing stress responses, and posing a serious threat to aquatic animals and, subsequently, human health.

## 3. Stress Responsiveness of MPs in Water Environment on Aquatic Animals

### 3.1. Stress of MPs through Food Chain

In size, shape, and color, MPs as an environmental stressor resemble plankton, which makes it difficult for small fish and invertebrates to distinguish MPs from food [4,8]. As shown in Table 2, 48 *Procambarus clarkii* from a pond breeding station and rice-crayfish co-culture breeding station in Hubei in China [18], 6 fish species in Han River and its tributaries in South Korea [28], 38 of 424 fish in the northern Baltic Sea [30], bivalve in Korea [35], 7 of the popular shellfish bought from the largest fish markets in Qingdao and Xiamen in China [44], 9 species of zooplankton taxa in Bohai Sea and Yellow Sea [45], 60 species of the freshwater fish *Squalius cephalus* in the Marne and Seine rivers around Paris [46], 27 species of marine and freshwater fish from the South China Sea, the East China Sea, the Yangtze River Estuary and Tai Lake [47], 73 of the 150 examined fish in Northwest Portuguese coastal waters, North East Atlantic Ocean [48], 32 fish species from Zhanjiang mangrove wetland, except *Atherina bleekeri* and *Stolephorus commersonnii* [49], and 13 of 17 commercial fish species in the north-eastern Mediterranean Sea [50] were found to have MPs in their body. Furthermore, fish meal is an important exposure route of MPs for aquatic animals. A study reported that MPs were detected in salmon, sardine, and kilka collected from the Persian Gulf and Caspian Sea, and also in all cultured *Cyprinus carpio* fed with commercial fish meal made of the above fish [51]. The abundance of MPs in fish meal obtained from Chilean wild catch anchovies [52], and produced in the United States, Denmark, Myanmar, Mauritania, Mexico, Peru, Panama, Chile, China, and Russia [53] was 50–100 mg/kg and 5.5 ± 1.6 items/g, respectively. The existing research further indicates that MPs as a stressor can cause enrichment in vivo and transfer through food chain.

Table 3 illustrates the studies on stress responsiveness of MPs in aquatic animals in the laboratory. It has been demonstrated that MPs can accumulate in vivo for a much longer time to cause stress responsiveness, such as by affecting the growth and development [59,60,61], body compositions [62], intestinal flora [63,64,65,66,67], immune system [68,69], and reproductive system [70], and causing oxidative stress [71,72,73,74] and histopathology [75,76] in aquatic animals. As MPs accumulated in the aquatic animals due to continuous ingestion, the survival rate, body length of *Chironomus tepperi* larvae, and adult emergence rate of the sediment-dwelling invertebrates were reported to decrease [77]. As a stressor, MPs not only affected the feeding behavior and life cycle of *Artemia salina* [78], but also caused oxidative stress by inducing reactive oxygen species (ROS) production and disturbing the antioxidant biomarkers. This impacted on mortality in nauplii and meta nauplii, changed swimming behavior, and damaged epithelial cells of juvenile *Artemia salina* [79]. Bioaccumulation, apoptosis, and several ROS-medicated dysfunctions were observed in brine shrimp after acute and chronic MP stress exposure [80], indicating that MPs can induce stress responsiveness. Furthermore, MPs affected the changes in behavior (e.g., average distance between fish, swimming speed, hunting behavior, and exploration ability), caused respiration stress, and interfered with ammonia excretion in juvenile *Sebastes schlegelii* [62], depressed immunity and produced oxidative stress in *Dicentrarchus labrax* L. [68]. They also increased levels of TNF-α, IFN-γ, TRl4, and IL-6 proteins in the gut of juvenile *Poecilia reticulata* [69], and affected the activity of acid phosphatase (ACP), alkaline phosphatase (AKP), lysozyme (LSZ), and phenoloxidase (PO) in the hepatopancreas and hemolymph in a time- and dose-dependent manner in response to MP stress in juvenile *Eriocheir sinensis* [64]. It has been reported that MPs could decrease reproductive capacity and early embryonic development, and cause oxidative stress by increasing malondialdehyde (MDA) levels in the gills, intestines, liver and gonads, inhibiting superoxide dismutase (SOD), glutathione S-transferase (GST) and catalase (CAT) activities, as well as reducing glutathione (GSH) and glutathione peroxidase (GSH-PX) contents in three-month old *Oryzias melastigma* [75], and inhibit the embryo hatchability of *Danio rerio* by 37% at 500 mg/L [70]. Additionally, MPs cause oxidative stress through the excessive production of ROS, which is an important mechanism in the toxic effects of MPs. In addition, photoaged MPs down-regulated genes including *cd36*, *mttp*, and *dgat2*, which are associated with triglyceride resynthesis and transportation to cause lipid malabsorption and growth inhibition in zebrafish larvae [81]. The MPs could accumulate total bile acids and change the nutrient metabolism in *Perca flavescens* [82], impair digestion and nutrient absorption functions in *Oreochromis urolepis* [83], and influence the digestive gene expression in *Oryzias latipes*, affecting nutrient absorption and antioxidant production [84].

However, a few studies reported that a significant fraction of MPs can be excreted out of the body [85,86,87], and MP stress did not affect the feeding and swimming behaviors in *Dicentrarchus labrax* L. [68], *Carassius auratus* larvae [61], and *Xenopus laevis* tadpoles [88]. No death or deformity was observed in juvenile *Oreochromis niloticus* under the environmental stress caused by MPs [89]. Similarly, no significant difference in body weight and length was noted in *Cyprinus carpio var*. larvae [90]. There inconsistent results were mainly affected by specific species and growth phase of aquatic animals (larvae, juvenile, or adult), time (acute or chronic), and method (exposure in water or in dietary) of experiment, and particle diameter, concentration, and type of MPs.

In summary, stress caused by MPs on aquatic animals is described. These findings motivate the needs for further research on their toxicity associated mechanism(s) which could provide possible solutions for pollution abatement.

### 3.2. Stress of Toxic Substances Released during Plastic Degradation Process

A variety of fillers, plasticizers, stabilizers, lubricants, and pigments are incorporated into plastics during the production process to enhance their performance. The additives in plastics may be released after long-term physical, chemical, or biological processes, increasing environmental stress. These processes produce the toxic intermediate products, most of which can cause reproductive toxicity, development deficiency, and death in aquatic animals [93]. Guo et al. (2019) reported that hexabromocyclododecanes (HBCDs) had adverse effects on thyroid hormone and oxidative stress levels of *Danio rerio* [94]. Phthalate ester (PAE)-based plasticizers, which have the characteristics of environmental stability, low degradation, and easy bioaccumulation [95], were detected in water (not detected—2.29 μg/L) and fish (not detected—1081 μg/kg dry weight) in the Asan Lake in Korea [96], as well as in Chlorella vulgaris and *Pagrosomus major* [97], which are able to cause irreversible damage, such as endocrine disruption and reproductive toxicity, to the human body through the food chain. Furthermore, di(2-ethylhexyl)phthalate (DEHP), a commonly-used plasticizer, significantly altered growth and locomotion by inducing oxidative stress, neurotoxicity and apoptosis pathways in *Oryzias latipes* [98], and caused immunotoxicity, reproductive toxicity and lipid metabolism disorder in *Oreochromis niloticus* [99]. In addition, bis(2-ethylhexyl)-2,3,4,5-tetrabromophthalate (TBPH) affected lipid metabolism in *Danio rerio* DNA methylation modification [100]. Moreover, plasticizers, such as bisphenol-A (BPA), released during the degradation of plastics, were reported to affect animal reproduction, hinder the development of crustaceans, and even induce genetic aberration [4]. Indeed, BPA could improve fatty acid synthetase, and uptake and suppression of β-oxidation, thereby inducing liver steatosis and endoplasmic reticulum stress in zebrafish [101]; it could also increase the micronuclei frequency of erythrocytes and plasma vitellogenin in *Salmo trutta*, causing endocrine disruption and genotoxic effects [102], and could disrupt testis maturation through apoptosis of germ cells and Leydig cells, influencing spermatogenesis in *Carassius auratus* [103]. The possible relationship between BPA-induced genotoxicity and oxidative stress might be mediated by changes in metabolic status [102].

### 3.3. MPs as Carriers of Multi-Stressors

The characteristics of MPs, such as small volume, large specific surface area, high hydrophobicity, and a strong affinity to heavy metals, enable them to easily interact with organic pollutants (e.g., polychlorinated biphenyls, polybrominated diphenyl ethers, and polycyclic aromatic hydrocarbons). In addition, MPs can also adsorb heavy metal pollutants, such as lead, zinc, and copper. Thus, MPs act as the carriers of many marine pollutants. It is important to note that MPs of different sizes and types exhibit variable adsorption of organic and heavy metal pollutants. For instance, the small-sized MPs have been reported to exhibit a strong adsorption capacity towards metal ions (0.261–0.579 mg/g) as compared to the large-sized MPs (0.243–0.525 mg/g), and the order of affinity of heavy metals to MPs is as follows: Pb^2+^ > Cu^2+^ > Cd^2+^ > Ni^2+^ [104]. Furthermore, the adsorption capacity of MPs towards antibiotics (such as oxytetracycline and ciprofloxacin) was significant, and the adsorption order is as follows: chlorinated polyethylene (CPE) > polyvinyl chloride (PVC) > polyethylene (PE) [105]. The density, aging degree, color, and crystallinity of MPs also affect their adsorption capacity towards organic and heavy metal pollutants [106]. Table 4 illustrates the related studies on the comprehensive stress of MPs and heavy metals as stressors on aquatic animals. The heavy metals (copper, lead, and cadmium) attached to MPs caused oxidative stress (such as lipid peroxidation) and enhanced mortality in *Hippocampus kuda* [107]. In addition, there was a statistically significant interaction between cadmium (Cd) and MPs on biochemical (such as AST, ALT, and ALP) and immunological (such as complement C3, total immunoglobulins, and lysozyme) parameters of *Cyprinus carpio* [108], as well as AChE activity in juvenile *Pomatoschistus microps* [109]. The interaction between MPs and Cd also reduced the number of hatched eggs and nutrient levels in newborn cladoceran *Moina Monogolica Daday*, negatively impacting their reproduction [110]. It has been reported that the accumulated Cd, oxidative stress, and inflammation in *Danio rerio* [111], and lethal and sublethal toxicity effects on the *Danio rerio* embryos [112] were observed after MP and Cd combined exposure. Furthermore, Cd accumulation, CAT activity, and GSH levels in juvenile *Symphysodon aequifasciatus* were affected by MPs and Cd, while, in the interaction between the two stressors, MT level was affected by Cd and the interaction between MPs and Cd [113]. Combined exposure to MPs and Cd induced significant hepatic stress and inflammation in *Cyprinus carpio* [114], and induced oxidative stress, apoptosis, and disturbance of the GH/IGF axis in the early life stages of zebrafish [115]. However, due to limited research on combined exposure to MPs and Cd on aquatic animals, it is necessary to investigate the complex mechanisms of the joint toxicity of MPs with other contaminants.

Additionally, the porous surface structure of MPs also provides an optimal habitat for colonization, diffusion, the alimentary cycle, and biofilm formation [117]. Biofilm formation on MPs is mainly divided into three processes, namely primitive colonization, growth, and maturation. Firstly, bacteria colonize on the surface of MPs and interact with the regulatory membrane. Secondly, they form irreversible attachments through the formation of fimbriae, adhesion proteins, and extracellular polymeric substances. Finally, microorganisms further proliferate and mature [118]. A study identified and characterized, for the first time, fungal genera on plastic debris which were collected at a harbor and an offshore location in the Belgian part of the North Sea [119]. Filaments, bacteria, and diatom-like structures were found in MP biofilms in three lakes in Northeastern Germany [120]. *Cyanobacteria* were particularly overrepresented in plastic marine debris, collected during the TARA Mediterranean expedition, together with essential functions for biofilm formation and maturation [121]. A study has showed that obvious microbial aggregates, mainly bacilli and cocci (shape), were observed on the surfaces of four types of MPs, namely PE, polystyrene (PS), polylactic acid (PLA), and PVC, after 45 days of exposure to the urban mangrove system in Hainan University, and the total amount of surface biofilm order is as follows: PE > PLA > PS > PVC [122]. In addition, *Bacillus subtilis* exhibited greater biofilm formation than *Staphylococcus aureus*, with submicron-sized and conventional plastics [123]. The diversity and abundance of cultivable antibiotic resistant bacteria in MPs, which were collected in an industrial mariculture system in Yantai city, were high, with the predominant bacterial genera of *Vibrio*, *Muricauda* and *Ruegeria*. In multi-antibiotic resistant bacteria (MARB) isolates, the positive detection rate of antibiotic resistance genes was up to 80.0% in MPs, while it was 65.3% in water, suggesting that MPs served as the hotspot for MARB and as a carrier for the spread of antibiotic resistance [124]. Half of the top 20 most abundant genera colonizing low-density polyethylene (LDPE) in lake water were found to be potential pathogens (e.g., plant pathogens *Agrobacterium*, nosocomial pathogens *Chryseobacterium*, and fish pathogens *Flavobacterium*) [125]. Furthermore, microorganism-colonized MPs are able to produce extracellular polymeric substances (EPS) that can facilitate heavy metal adsorption from surrounding water [126], seriously threatening aquatic animal health. The microbial community structure on the surface of MPs is often different from that of surrounding water environment, and it can be released into the water environment and alter the existing ecosystem. Meanwhile, the combination of MPs, biofilm, and heavy metal increases the risk and stress to the environment. Therefore, characteristics of microbial community on the surface of MPs need to be studied further in order to provide an important reference for further ecological risk assessment of MP stress.

## 4. Summary and Future Prospects

As plastic disposal and sewage treatment technologies have still not yet been optimized, MPs have become a focus of social and environmental concern. Currently, the research efforts on MPs worldwide mainly focus on the stress of MPs on aquatic animals. The prevention and control of plastic pollution at its origin is the best way to safeguard aquatic organisms. The following aspects are needed to strengthen this in the future.

Laws and regulations, along with effective enforcement, are fundamental. Necessary regulation includes implementing and promoting a ban on plastics, enforcing the classification and recycling of plastic waste, improving the sewage treatment technology, and regularly cleaning water bodies.

The plastic industry must abide by the laws and regulations and must standardize the application and treatment of plastic products along with other environmental-friendly business practices. For example, biodegradable plastic substitutes are needed to be developed urgently. The industrial plastic waste must not be randomly discarded, and the enterprises must uptake mutual supervision.

The environmental awareness of the public should be improved through education programs. The public should consciously commit to garbage sorting, avoid (or reduce) the use of disposable plastic products, and encourage friends and family to strictly adhere to any plastic ban.

## Figures and Tables

**Table 1:** The ubiquitous distribution of MPs as a stressor in global water bodies.

Location	Particle Size	Main Types	Abundance	Reference
Two large municipal treatment plants (Shanghai, China)	80–5000 μm	PET, PA, PE, PP	226.27 ± 83.00 and 171.89 ± 62.98 piece/L, respectively (influent water of WWTP1 and WWTP2)	[10]
Two municipal wastewater treatment plants (Harbin, China)	0.038–0.55 mm	PP, PS, PE	Approx. 1.47 × 10^6^ particles (the daily emission)	[11]
Dongting Lake, Hong Lake (China)	50–5000 μm	PE, PP, PS, PVC	900–2800 (Dongting Lake) and 1250–4650 (Hong Lake) n/m^3^	[12]
Three Gorges Reservoir (China)	112 μm–5 mm	PE, PP, PS	3407.7 × 10^3^ to 13,617.5 × 10^3^ (in the mainstream) and 192.5 × 10^3^ to 11,889.7 × 10^3^ (the tributaries) items/km^2^	[13]
Yangtze Estuary System (China)	0.90 ± 0.74 mm (Yangtze Estuary), 2.01 ± 2.01 mm (East China Sea)	/	4137.3 ± 2461.5 (estuary) and 0.167 ± 0.138 (sea) n/m^3^	[14]
Haizhou Bay (China)	0.08–13.48 mm (surface water), 0.04–14.74 mm (sediment)	Rayon, PET, PP, PE	2.60 ± 1.40 items/m^3^ (surface water) and 0.33 ± 0.26 (sediment) items/g	[15]
Urban surface waters (Wuhan, China)	<2 mm accounted for >80%	PET, PP, PE, PA, PS	1660.0 ± 639.1 to 8925 ± 1591 n/m^3^	[16]
Pearl River Estuary and Guangzhou urban area (China)	0.05–5 mm	PA, PVC cellophane, PP, PE, VACs	19,860 items/m^3^ (urban section), 8902 items/m^3^ (estuary)	[17]
A pond rice-crayfish co-culture water and sediments (Hubei, China)	<1 mm accounted for 42.8 ± 20.8% to 89.1 ± 5.0% (water) and 58.8 ± 16.7% to 97.0 ± 4.8% (sediment)	PP:PE, PE, cellulose, cellophane, PET	1.3 ± 0.1–2.5 ± 0.1 particles/L (water), 0.03 ± 0.01–0.04 ± 0.02 particles/g (sediment)	[18]
Elbe water and sediments (Europe)	150–5000 μm (water), 125–5000 μm (sediment)	PE, PP, PS ABS, PA, PET, PMMA	5.57 (water) and 3.35 × 10^6^ (sediment) particles/m^3^	[19]
Lake Winnipeg (Canada)	<5 mm	/	193,420 ± 115,567 particles/km^2^	[20]
The center of the Mediterranean Sea	<3 mm (73%)	PE (69%), PP (24%), PS, PET	0.01 to 0.66 particles/m^2^	[21]
Temperate waters north of the Subtropical Front to the Southern Ocean	<5 mm (93%)	PE, PP, PS, PVC, PA, PMMA	7.7–14.1 (temperate waters), 1.1–2.6 (Southern Ocean) g/km^2^	[22]
Pacific Northwest	0.5–1.0 mm (~50%), 1–2.5 mm (29.8%), 2.5–5.0 mm (17.6%)	PE, PP, PA, PVC, PS, rubber, PET	6.4 × 10^2^ to 4.2 × 10^4^ items/km^2^	[23]
The mid-west Pacific Ocean	0.3–2.5 mm	PP, PMMA, PE, PET	6028–95,335 pieces/km^2^	[24]
Persian Gulf	100–5000 μm	PE, PP, PS	1.5 × 10^3^ to 4.6 × 10^4^ particle/km^2^	[25]
The lower Chao Phraya (Thailand)	0.05–1.0 mm (surface water), 0.05–0.3 mm (sediment)	PP, PS, PE	80 ± 65 items/m^3^ (surface water), 91 ± 13 items/kg (sediment)	[26]
Ems River (Germany)	/	PE	1.54 ± 1.54 items/m^3^	[27]
Han River (South Korea)	<1 mm accounted for >90%	Silicone, PE, PS, PTFE, polyester	7.0 ± 12.9 (surface), 102.0 ± 50.3 (2 m below the surface), 91.1 ± 72.3 (the tributaries) particles/m^3^	[28]
The nearshore water of South Georgia	50–100 μm (38.1% in seawater, 45.5% in freshwater, 46.7% in wastewater)	PET	2.67 ± 3.05 MPs/L (freshwater), 4.67 ± 3.21 MPs/L (precipitation), 1.66 ± 3.00 MPs/L (wastewater)	[29]
The northern Baltic Sea (Finland)	140–2370 μm	/	16.2 ± 11.2 MPs/m^3^	[30]
Southern Caspian Sea	1000–5000 μm	PE, PP, PET	0.246 ± 0.020 MPs/m^3^	[31]
Surface water in Rize (Turkey)	/	PA, PET	1–13 items/L	[32]
Rawal Lake (Pakistan)	/	PE, PP, polyester, PET, PVC	0.142 items/0.1 L (water), 1.04 items/0.01 kg (sediment)	[33]
Terengganu estuary and offshore (Malaysia)	/	PA, PE, PP	1687 particles/m^3^ (estuary), 1900 particles/m^3^ (offshore)	[34]
Along the Korean coasts	<300 µm	PP, PE, and polyester	1400 ± 560 n/m^3^	[35]
Marine Protected Areas of Southern Sri Lanka	3.14 ± 0.17 and 2.96 ± 0.18 mm (Bundala National Park), 3.46 ± 0.16 and 3.39 ± 0.43 mm (Hikkaduwa Marine National Park)	PE, PP, PS	0.515 ± 0.054 MPs/m^3^ (Hikkaduwa Marine National Park), 0.276 ± 0.077 MPs/m^3^ (Bundala National Park)	[36]
Suburban areas, plain countryside and mountainous areas (Zhejiang, China)	>0.1 mm (West Lake District-1), 0.0308–0.1 mm (the rest of areas)	PP, PS, PET, PES, PE, PA	430–540 items/m^3^ (suburban countryside) and 1150–2154 items/m^3^ (plain countryside and mountainous areas)	[37]

Abbreviations are as follows: PE = polyethylene, PP = polypropylene, PS = polystyrene, PVC = polyvinylchloride, PET = polyethylene terephthalate, PA = polyamide, VACs = vinyl acetate copolymers, PES = polyethersulfone, PP:PE = poly(propylene–ethylene) copolymer, ABS = acrylonitrile butadiene styrene, PMMA = polymethyl methacrylate, PTFE = polytetrafluoroethylene.

**Table 2:** Characteristics of MPs found in fish and fish meal.

Species	Particle Size	Shape	Abundance	Reference
*Procambarus clarkii*	50 to 500 μm	Fiber and fragments	0.17 ± 0.07–0.92 ± 0.19 particles/individual	[18]
*Cyrinus carpio*, *Carassius cuvieri*, *Lepomis macrochirus*, *Micropterus salmoides*, *Silurusasotus*, *Channa argus*	0.3–0.6 mm	Fragments (>94%) and fiber	4–48 particles/fish (intestine), 8.3 ± 6.0 particles/fish (gill)	[28]
*Three-spined stickleback*, Bleak, Perch, Roach	184 to 2592 μm	/	51 MPs in 38 fish/424 fish	[30]
Calanoida, Cyclopoida, Harpacticoida, Mysids, Decapoda, Cladocera	68–144 µm and 400.1–500 µm (offshore), 80–99 µm (estuary)	Fiber and fragments	0.01 ± 0.002 to 0.20 ± 0.14 particles/individual	[34]
Oyster, mussel, Manila calm	<300 µm	Fragments	1.21 ± 0.68 n/individual (Oyster/mussel), 2.19 ± 1.20 n/individual (Manila calm)	[35]
Mussel, *Ruditapes*, *philippinarum*, *Crassostrea gigas*, *Sinonovacula constricta*, *Scapharca subcrenata*, *Meretrix lusoria*, *Busycon canaliculatu*	983.8 μm (from Qingdao), 1011.2 μm (from Xiamen)	Fiber, granules, film, and fragments	1.2–4.1 (Qingdao) and 1.3–6.0 (Xiamen) items/individual	[44]
Copepoda, Macrura, Brachyuran, Amphipoda, Cumacea, Lernaea, Scaleph, Larval fish, Tunicata	0.1–1 mm	Fiber (81.53%), fragments (11.71%), line (4.95%), and particles (1.81%)	0.06–4.55 particles/m^3^	[45]
*Squalius cephalus*	2.41 mm	Fiber and fragments	18 APs in 60 stomachs and 5% livers contained one or more APs	[46]
*Hyporhamphus intermedius*, *Liza haematocheila*, *Coilia ectenes*, *Lateolabrax japonicas*, *Sillago sihama*, *Larimichthys crocea*, *Psenopsis anomala*, *Pampus cinereus*, *Harpodon nehereus*, *Mugil cephalus*, *Muraenesox cinereus*, *Terapon jarbua*, *Sebastiscus marmoratus*, *Photopectoralis bindus*, *Cynoglossus abbreviates*, *Thamnaconus septentrionalis*, *Oxyeleotrix marmorata*, *Synechogobius ommaturus*, *Collichthys lucidus*, *Branchiostegus japonicas*, *Callionymus planus* *Cyprinus carpio*, *Carassius auratus*, *Hypophthalmichthys molitrix*, *Pseudorasbora parva*, *Megalobrama amblycephala*, *Hemiculter bleekeri*	0.04–5 mm (MPs) and 5.1–24.8 mm (mesoplastics)	Fiber, fragments, and film	1.1 to 7.2 items/individual (MPs), 0.2 to 3.0 items/individual (mesoplastics)	[47]
*Dicentrachus labrax*, *Trachurus trachurus*, *Scomber colias*	501–1500 µm (gastrointestinal tract) and 151–500 µm (gill)	Fiber (54%), fragments (45%), and pellets (1%)	1.2 ± 2.0 items/individual (gastrointestinal tract), 0.7 ± 1.2 items/individual (gill) and 0.054 ± 0.099 items/g (dorsal muscle)	[48]
*Amoya chlorostigmatoides*, *Acanthopagrus latus*, *Arius leiotetocephalus*, *Acentrogobius viridipunctatus*, *Cynoglossus puncticeps*, *Callionymus richardsoni*, *Dendrophysa russelii*, *Epinephelus akaara*, *Gerreomorpha decacantha*, *Gerres filamentosus*, *Gerres lucidus*, *Muraenesox cinereus*, *Oreochromis niloticus*, *Odontamblyopus rubicundus*, *Pisodonophis boro*, *Platycephalus indicus*, *Periophthalmus modestus*, *Solea ovate*, *Sillago sihama*, *Terapon jarbua*, *Takifugu niphobles*, *Zebrias zebra*, *Atherina bleekeri*, *Alepes djedaba*, *Caranx malam*, *Konosirus punctatus*, *Leiognathus brevirostris*, *Osteomugil ophuyseni*, *Osteomugil stronylophalus*, *Stolephorus commersonnii*, *Tylosurus melanotus*, *Thryssa vitrirostris*	0.02–5 mm	Fiber (70%), fragments (18%), film (9%), and pellets (3%)	0.6 to 8.0 items/individual	[49]
*Boops boops*, *Dentex macrophthalmus*, *Dentex maroccanus*, *Lepidotrigla cavillone*, *Mullus barbatus*, *Saurida lessepsianus*, *Trigla lucerna*, *Upeneus moluccensis*, *Upeneus pori*, *Pagellus erythrinus*, *Etrumeus golanii*, *Sardina pilchardus*, *Trachurus mediterraneus*	1.26 ± 1.38 mm	Film, fragments, pellets, and rubber	1.3 MPs/individual	[50]
*Cyprinus carpio*	452 ± 161 µm	Fragments (65%), film (25%), pellets (7%), and fiber (3%)	57 MPs/150 fish	[51]
Anchovy	/	/	50–100 mg/kg	[52]
Six batches of fish meal produced from the United States, Denmark, Myanmar, Mauritania, Mexico, Chile, Peru, Panama, China, and Russia purchased from Huangyan Ensor Feed Corporation (China)	500–1000 µm	Fiber	5.5 ± 1.6 items/g	[53]
Two kinds of fish meal comprised of fish mainly *Rastrelliger kanagurta*, and one kinds of fish meal comprised of fish waste	855.82 ± 1082.90 µm	Fragments (78.2%), filaments (13.4%), and film (8.4%)	216 particles/336 particles (64.3%)	[54]
Twenty-six kinds of fish meal produced from Antarctica, Chile, China, Denmark, India, Morocco, Mauritania, Norway, Peru, South Africa, South Korea, and Turkey	4.2 ± 0.3 mm	Fiber, film, and fragments	8.9 ± 1.0 particles/50 g	[55]
Sixteen commercially available angling baits products (six groundbaits, six boilies, and four pellets)	700 µm–5 mm	Fragments	17.4 MPs/kg (groundbaits), 6.78 mg/kg (boilies), not detected (pellets)	[56]
*Oreochromis niloticus*, *Prochilodus magdalenae*, *Pimelodus grosskopfii*	/	Fragments, film, and fiber	2.1 ± 1.26 items/individual	[57]
*Sparus aurata*, *Cyprinus carpio*	0.24 to 8.86 mm (*S. aurata*), 0.07 to 2.23 mm (*C. carpio*)	Fiber (*S. aurata*), fiber and fragments (*C. carpio*)	0.48 items/individual (fry and adult *S. aurata*), 0.11 items/individual (fry and adult *C. carpio*)	[58]

Here, Aps = Anthropogenic particles.

**Table 3:** The studies on stress responsiveness of MPs on aquatic animals in laboratory.

Model Animal	Particle Size	MP Concentration	Time	Toxicological Effects	Reference
*Sparus aurata*	/	3.33 g/kg of feed	45 d	Did not induce stress, pathology, accumulate in the gastrointestinal tract and alter the growth rate	[58]
*Danio rerio*	PA, PE, PP, PVC: ~70 μm and PS: 0.1, 1.0, 5.0 μm	0.001–10.0 mg/L	10 d	Enhanced mortality and histopathology	[59]
*Scophthalmus Maximus*	20 μm	0, 100, 200 and 300 particles/L	46 d	Enhanced mortality	[60]
*Carassius auratus*	70 nm and 50 μm	10, 100 and 1000 μg/L	7 d	Bioaccumulation, oxidative stress, tissue (intestine, liver and gill) damage, inhibiting growth and swimming speed	[61]
*Sebastes schlegelii*	0.5 and 15 μm	190 μg/L	14 d	Histopathology, altered behavior, energy reserve and nutritional quality, enhanced mortality, respiration and metabolism stress	[62]
Adult *Danio rerio*	8 μm	10 μg/L and 1 mg/L	21 d	Caused microbiota dysbiosis and inflammation	[63]
*Eriocheir sinensis*	5 μm	0, 0.04, 0.4, 4 and 40 mg/L	21 d	Affected non-specific immune responses and intestinal microflora	[64]
*Danio rerio*	5 and 50 μm	100 and 1000 μg/L	7 d	Intestinal flora, oxidative stress, immune response, neurodevelopment, swimming behavior, hepatic metabolism, growth and metabolism	[65]
*Danio rerio*	5 μm	50 and 500 μg/L	21 d	Inflammation, oxidative stress, altered in gut metabolome and microbiome	[66]
*Larimichthys crocea*	100 nm	0, 10, 10^4^ and 10^6^ items/L	14 d	Enhanced mortality, reduced immune and digestive enzyme activities and caused microbiota dysbiosis	[67]
*Dicentrarchus labrax* L.	104.1 ± 36.2 µm (PVC), 77.5 ± 18.3 µm (PE)	100 or 500 mg PVC or PE kg^−1^ diet	3 w	Oxidative stress, immune response, and histopathology	[68]
*Poecilia reticulata*	33–40 μm	100 and 1000 μg/L	28 d	Impaired digestive performance, stimulating immune response, and inducing microbiota dysbiosis	[69]
*Danio rerio*	0.5 and 10 μm	0.1, 1.0, 10, 100, 200 and 500 mg/L	3 d	Enhanced mortality and embryo hatching rates	[70]
*Danio rerio*	70 nm, 5 μm and 20 μm	20, 200 and 2000 μg/L	7 d	Inflammation, altered of metabolic profiles, oxidative stress, lipid accumulation	[71]
Yellow River Carp	100–200 μm	10%, 20%, 30%, 40% PVC in diets	60 d	Gonadal development, oxidative stress, and immune function	[72]
*Danio rerio*	5 and 50 μm	100 and 1000 μg/L	7 d	Oxidative stress, altered of metabolic profiles, glycolysis-related and lipid metabolism-related genes	[73]
Red Crucian Carp	124 μm	150, 300 and 600 μg/L	96 h	Oxidative stress	[74]
*Oryzias melastigma*	10 μm	2, 20 and 200 μg/L	60 d	Oxidative stress, histopathology, decreased fecundity, altered the HPG	[75]
*Danio rerio*	15 μm	20 mg/L	24 h	Histopathology, inflammation, metabolism disruption, gut microbiota dysbiosis and bacteria alterations	[76]
Sediment-dwelling invertebrate	1–4, 10–27, 43–54 and 100–126 μm	500 per kg sediments	10 d	Enhanced mortality, body length, head capsule and emergence	[77]
*Artemia salina*	10 μm	1, 10, 100, 1000, and 10,000 MPs/mL	7 d	Affected feeding behavior and life cycle	[78]
*Artemia salina*	11.86–44.62 μm	1, 25, 50, 75 and 100 μg/mL	48 h	Accumulation, oxidative stress, changed swimming behavior, histopathology	[79]
Brine shrimp	5 μm	1, 25, 50, 75 and 100 mg/L	14 d	Generation of ROS, histopathology, and transcriptome	[80]
*Danio rerio*	~32.50 μm	1, 10, 20 mg/L	2 hpf–10 dpf	Growth inhibition, intestine injury, and lipid malabsorption	[81]
*Perca flavescens*	100–125 μm	0, 1, 2, 4 and 8 g MPs/100 g dry diet	9 w	Changed nutrient metabolism, decreased nutritional fish quality, disrupted intestinal histopathology and microbiota diversity	[82]
*Oreochromis urolepis*	38–45 μm	1, 10, 100 MPs/mL	65 d	Growth inhibition, intestine damage, impaired digestion and nutrient absorption functions	[83]
*Oryzias latipes*	100 (larvae) and 400 (juvenile) μm	0.5, 1.5, 3 and 6 MPs/fish/day	21 d	Influenced digestive gene expression	[84]
*Danio rerio*	10–20, 45–53, 250–300 μm	3 × 10^4^ particles/L	12 h, 4 w	The elimination and distribution of MPs presented size-effect and time-effect relationships	[85]
*Gobiocypris rarus* larvae	0.1, 1, 10 μm	0.055, 0.55, 5.5, 55 and 550 μg/L	7 d	No observed effect	[86]
*Tigriopus japonicus*	10 μm	1 × 10^3^ particle/mL	0, 3, 6, 9, 12, 24, 48 h	Accumulation	[87]
*Xenopus laevis*	3 μm	0.125, 1.25 and 12.5 μg/mL	Stage 36 to stage 46	Neither body growth nor swimming activity were affected	[88]
*Oreochromis niloticus*	0.1 μm	1, 10 and 100 μg/L	14 d	Neurotoxicity and oxidative stress	[89]
*Cyprinus carpio var.*	/	10%, 20%, 30% MPs in diets	60 d	Oxidative stress, antioxidant-related gene, growth, and histopathology	[90]
*Carassius auratus*	0.7–5.0 mm (fiber), 2.5–3.0 mm (fragments), 4.9–5.0 mm (pellets)	0.96%, 1.36%, 1.94%, 3.81% (g(food + MPs)/g fish)	6 w	Observed sub-lethal effects, weight loss, histopathology, and inflammation	[91]
*Sebastes schlegelii*	15 μm	1 × 10^6^ particle/L	14 d	Weakened feeding activity and hunting behavior, histopathology, and influenced the energy reserve and nutritional quality	[92]

Abbreviations are as follows: PA = polyamide, PE = polyethylene, PP = polypropylene, PVC = polyvinylchloride, PS = polystyrene, HPG = hypothalamus-pituitary-gonadal, ROS = reactive oxygen species, hpf = h post fertilization, dpf = days post fertilization.

**Table 4:** Related studies on the comprehensive stress of MPs and heavy metals on aquatic animals.

Model Animal	Concentration	Time	Toxicological Effects	Reference
*Hippocampus kuda*	MPs (15–80 μm): 0.1 g/3 L, copper: 0.05 mg/L, Cd: 0.01 mg/L, lead: 0.05 mg/L	45 d	Growth, enhanced mortality, and oxidative stress	[107]
*Cyprinus carpio*	MPs: 250 and 500 µg/L, Cd: 100 and 200 µg/L	30 d	Altered biochemical and immunological parameters	[108]
*Pomatoschistus microps*	MPs (1–5 μm): 0.18 mg/L, Cd: 3, 6, 13, 25 and 50 mg/L	96 h	Observed sub-lethal effects, especially neurotoxicity	[109]
*Moina monogolica Daday*	MPs (2–4 μm): 300 µg/L, Cd: 5 and 10 µg/L	21 d	Enhanced mortality, poor nutritional status in progeny, MPs with adsorbed Cd showed greater adverse dose-dependent effects	[110]
*Danio rerio*	MPs: 20 mg/L, Cd: 100 mg/L	3 w	Oxidative stress, inflammation, histopathological, affected functional gene expression, and increased accumulation of Cd	[111]
*Danio rerio*	PS: 0.05, 0.1, 1, 5, 10 mg/L, Cd: 0.01 mg/L	96 h	Negative impacts on survival and heart rate, observed lethal and sublethal effects	[112]
*Symphysodon aequifasciatus*	MPs (32–40 μm): 50 and 500 µg/L, Cd: 25 and 50 µg/L	30 d	Reduced Cd accumulation, oxidative stress, stimulated innate immunity	[113]
*Cyprinus carpio*	MPs: 0.5 mg/L, copper: 0.25 mg/L	14 d	Facilitated copper accumulation, induced significant hepatic stress and inflammation	[114]
*Danio rerio*	MPs: 500 µg/L, Cd: 5 µg/L	30 d	Negative effects on growth, oxidative stress, and apoptosis	[115]
*Danio rerio*	MPs: 20 mg/L, Cd: 1 mg/L	4 hpf–120 hpf	Oxidative stress promoting taurine metabolism and unsaturated fatty biosynthesis	[116]

Here, Cd = cadmium.
